# Research gaps in viral hepatitis

**DOI:** 10.1002/jia2.25054

**Published:** 2018-04-10

**Authors:** Anders Boyd, Léa Duchesne, Karine Lacombe

**Affiliations:** ^1^ INSERM UMR_S1136 Institut Pierre Louis d'Epidémiologie et de Santé Publique Paris France; ^2^ Department of Infectious Diseases, Research and Prevention Public Health Service of Amsterdam Amsterdam Netherlands; ^3^ Department of Infectious and Tropical Diseases Saint‐Antoine Hospital AP‐HP Paris France; ^4^ Sorbonne Universités INSERM UPMC Univ Paris 06 Institut Pierre Louis d’épidémiologie et de Santé Publique (IPLESP UMRS 1136) Paris France

**Keywords:** hepatitis B virus, hepatitis C virus, public health, testing, antivirals, eradication

## Abstract

**Introduction:**

The World Health Organization has aimed for global elimination of both hepatitis B virus (HBV) and hepatitis C virus (HCV) by 2030. Treatments available to cure HCV and control HBV, as well as vaccination to prevent HBV infection, have certainly allowed for such bold goals, yet the final steps to usher in elimination require further evidence.

**Discussion:**

We broadly discuss the needs for three major public health approaches. First, an effective vaccine exists for HBV and mass‐vaccination campaigns have resulted in decreases in hepatitis B surface antigen seroprevalence and overall rates of liver‐related morality. Still, HBV vaccination coverage is poor in certain regions of the world, while the reasons for such low coverage require further study. A prophylactic vaccine is probably needed to eliminate HCV, but is not being readily developed. Second, identifying HBV/HCV infected individuals remains a priority to increase awareness of disease status, particularly for key populations. Research evaluating large‐scale implementation of novel, rapid and mobile point‐of‐care tests would be helpful to determine whether increased awareness is achievable in these settings. Third, antiviral therapy allows for strong HBV suppression and HCV cure, while its access depends on financial factors among many others. Although there is strong evidence to treat key populations and specific groups with progressed disease, as stated in current guidelines, the advantages of extending treatment eligibility to decrease onward spread of HBV/HCV infection and prevent further burden of disease are lacking “real world” evidence. Novel anti‐HBV treatments are being developed to target intrahepatocellular HBV replication, but are still in the early phases of clinical development. Each of the strategies mentioned above has specific implications for HIV infection.

**Conclusions:**

There are certainly effective tools to combat the spread of viral hepatitis and treat infected individuals – yet how they are able to reach key populations, and the infrastructure required to do so, continue to represent the largest research gap when evaluating the progress towards elimination. Continuously adapted and informed research is required to establish the priorities in achieving elimination goals.

## 1 Introduction

The 2015 update of the Global Burden of Diseases study has ranked chronic viral hepatitis and its underlying conditions, such as cirrhosis and liver cancer, among the top 20 causes of death, steeply increasing from 1990 to 2015 1. Nevertheless, an extensive array of tools is available to combat hepatitis B virus (HBV) and hepatitis C virus (HCV). HBV vaccination is effective at preventing infection. Individuals with chronic HBV‐infection are able to reduce circulating HBV with almost no risk of developing resistance when using the potent nucleoside/nucleotide analogues (NA) entecavir, tenofovir disoproxil fumarate (TDF), or the more recent tenofovir alafenamide 2. Novel direct‐acting antivirals (DAAs) have allowed for short‐term and effective treatment against HCV and are known to induce high rates of sustained virological response (SVR) 3.

With these effective means at hand, the World Health Organization (WHO) has aimed to achieve global elimination of both infections by 2030. However, elimination implies strengthening existing tools – more efficient screening policies, wider access to care and management of liver disease, and antiviral agents that ultimately halt the virus. In this commentary, we broadly highlight some recent advances, and room for improvement, in three public health strategies geared towards HBV and HCV elimination that also include HIV coinfected individuals: vaccination, testing and treatment.

## 2 Discussion

### 2.1 Preventing ongoing transmission

With an effective HBV vaccine, many large‐scale vaccination campaigns have been able to dramatically change the spectrum of liver‐associated disease. For instance, widespread vaccination in China has led to substantial decreases in incident HBV infections and the prevalence of hepatitis B surface antigen (HBsAg)‐positive serology 4. Moreover, recent data from Taiwan have confirmed that immunization in infants reduces the risk of hepatocellular carcinoma (HCC) during adulthood 5. Worldwide three‐dose HBV vaccine coverage has increased to 84% 6, yet a considerable proportion of the global population could still benefit from vaccination 7. Understanding the logistic, financial, and cultural constraints to vaccine access would help further the use of this highly‐effective, preventative measure.

Timely birth‐dose vaccination (<24 hours after birth) among infants born to HBsAg‐positive mothers has shown to prevent mother‐to‐child transmission. Birth‐dose coverage is unfortunately lagging at <40% for many countries, with the lowest coverage in Africa at 10% 6. As one‐fifth of infants vaccinated at birth and born to hepatitis B “e” antigen (HBeAg) positive mothers become infected 8, this strategy requires further improvement. Treating highly viraemic mothers with TDF has been explored as a strategy to decrease transmission rates. However, these studies have been limited to mostly Asia 9 and further evaluation is needed in other regions with higher liver disease burden, such as Africa.

No prophylactic vaccine is available for HCV infection, while the virus’ genetic variability and capacity to escape host immune responses make vaccine development a formidable challenge 10, 11. Recent findings on the viral envelope structure and innovative experimental animal models have uncovered new opportunities for HCV vaccine research 12. Nevertheless, there is ongoing debate whether HCV vaccination would even be useful towards elimination. Significant reduction in HCV incidence could be incurred from wide‐spread access to DAA in key populations 13, yet this “treatment‐as‐prevention” (TasP) approach has only been evaluated with models strongly dependent on HCV baseline prevalence, treatment coverage, transmission network structure, harm reduction uptake and post‐treatment changes in behaviour 14, 15. “Real‐world” evaluations could test the accuracy of these assumptions. Models of TasP have also demonstrated cost‐effectiveness among key populations 16. It remains unknown if TasP is still cost‐effective for other countries, especially those without voluntary licence agreements, where current DAA prices are prohibitive. Existing models have indicated that a vaccine, even if partially effective, could reduce HCV incidence at a lower cost than TasP 17, 18 and without requiring specialized care.

### 2.2 Identifying undiagnosed infection

#### 2.2.1 Infection unawareness

A targeted testing approach is not explicitly recommended for HBV 19, with the broad exception of testing all those “who do not know their status” 6, but is recommended for HCV based on risk or birth cohort 20. HIV infection is also identified as a risk factor due to shared routes of transmission 21, 22. With this in mind, the 2017 WHO Global Hepatitis Report estimated that an alarming 91% of HBsAg‐positive and 80% of HCV‐positive individuals were unaware of their infection 6. These estimates are considerably higher than the roughly 30% of HIV‐infected individuals unaware of their infection 23. Most undiagnosed cases of HBV and HCV in high‐income countries (HIC) belong to marginalized groups not covered by conventional screening programmes 20. Undiagnosed cases in low‐ to middle‐income countries (LMIC) are also found in these key populations, but the greater problem lies in limited centralized diagnostic facilities, cost of testing, lacking public education and awareness, and need for skilled health professionals 24, 25. Improving general understanding of viral hepatitis and its health consequences are also central to increasing disease awareness and could be addressed via public health campaigns 26.

#### 2.2.2 Novel rapid‐testing techniques

Despite the paucity of studies in LMIC, data support the cost‐effectiveness of HBV testing in key populations (i.e. migrants) for HIC and in general for low‐income countries with high HBsAg‐positive seroprevalence 7. Developing easy to use and affordable screening tools would allow a considerable scale‐up in testing. Transportable in size and straightforward in use, point‐of‐care (POC) systems could widen testing availability and specificity have been validated in both HIC and LMIC 27. Anti‐hepatitis B surface and anti‐hepatitis B core antibody status are also needed to determine HBV infection phase; however, rapid tests detecting these antibodies are either lacking or inadequate 27, 28.

PCR is the gold standard approach to evaluate viral replication. HCV RNA and HBV‐DNA are not often assessed due to high costs, heavy burden in human resources, which then restrains their availability 7. Some less‐costly molecular assays that could be easier to implement in LMIC are being developed, including a new semi‐quantitative real‐time PCR approach able to discriminate samples with HBV DNA levels above or below the clinically‐relevant threshold of 2000 IU/ml 29. However, field validation must be performed before concluding their usefulness in resource‐limited settings. POC assays could extend viral load testing and increase those aware of having active HCV‐infection. The stringent technical requirements to quantify HCV RNA offer little leeway for an inexpensive, portable POC test that must also be resistant to extreme environmental conditions and have a turnaround time of <1 hour. Systems have been developed, such as GeneXpert and Genedrive, that are able to accurately quantify HCV RNA and HBV‐DNA 30, 31, but the high cost of performing these assays limits its immediate appeal for LMIC. Other novel POCs targeting alternative markers of viraemic activity, such as Daktari and HCV core antigen detection 32, may pave the way for more affordable HCV POC tests. Next generation, microchip‐sized diagnostics have been emerging in which innovations in nanotechnology, bioengineering and microfluid dynamics are combined 33. Mobile tests using this technology could provide the low‐cost, accuracy, and fully‐automated features required for POC assays, yet are still in the early stages of development. In order to incorporate any of these diagnostic tools in screening guidelines, more comprehensive data on their accuracy, feasibility, acceptability and cost‐effectiveness would be needed.

### 2.3 Implications of antiviral therapy

#### 2.3.1 Need for antiviral treatment

The principle goal of NA treatment is suppressing HBV DNA to a level that is associated with reduced rates of liver‐related morbidity and mortality. Each phase of chronic HBV‐infection is associated with a varying degree of viral activity and risk of severe clinical outcomes. Accordingly, HBeAg‐positive or –negative chronic hepatitis B patients or those with compensate or decompensate cirrhosis are strongly recommended to initiate treatment 2. For other phases of infection, there is still debate on when to initiate treatment. Patients early on in infection (i.e. HBeAg‐positive chronic HBV infection) occasionally exhibit mild‐to‐severe liver fibrosis with highly active anti‐HBV T‐cell responses 34 and thus could benefit from early treatment. Other factors also drive the decision to treat, such as family risk of HCC (at high risk of disease) and older age (more willing to adhere to lifelong therapy) 35. In addition, certain patients (i.e. HBV mono‐infected individuals in sub‐Saharan Africa) are oftentimes not recommended for treatment based on most guidelines, but demonstrate increased risk of HCC 36. TDF‐containing antiretroviral therapy should be initiated as early as possible for all HIV‐HBV co‐infected patients 37, 38. Despite these recommendations, there is a dearth of clear, large‐scale evidence that delaying therapy affects HCC incidence or liver‐related mortality in these subgroups 35.

EASL practice guidelines have recently incorporated a section on stopping treatment 2, yet criteria for discontinuation remain rather ambiguous. It should be stressed that this practice is highly discouraged for HIV‐HBV co‐infected patients due to its association with severe clinical outcomes 39, 40. In brief, patients should discontinue treatment if they have sustained HBsAg‐loss. Treatment could be discontinued in (i) HBeAg‐positive chronic HBV patients who achieved HBeAg‐seroconversion, undetectable HBV‐DNA and normalized transaminases and underwent at least 12 months of consolidation therapy, and to a lesser extent, (ii) HBeAg‐negative patients with at least 3 years of virological suppression. Since these latter two groups have variable rates of virological and clinical relapse, close virological monitoring is strongly encouraged. Predicting those at risk of failing discontinuation is difficult considering that no available marker has the capacity to do so, with the possible exception of very low HBsAg titres 41. Whether other biomarkers, such as circulating HBV RNAs, could address, this issue remains speculative.

HCV treatment aims to cure infection and prevent further HCV‐associated complications. The most recent EASL guidelines recommend broadening access to DAA treatment, focusing on HCV‐infected individuals with significant fibrosis/cirrhosis, extrahepatic manifestations, HCV recurrence after liver transplantation, and risk of further transmission 3. These guidelines are applied similarly to HIV‐HCV co‐infected patients, yet special consideration should be given to drug‐drug interactions with other antiretroviral agents. Given the high SVR rates achieved with current regimens, there is strong advocacy for treating all HCV‐infected individuals. However, the cost burden of effective DAAs, especially for single‐payer healthcare systems, and its distribution remain obstacles for universal treatment 42.

Systematic reviews are available in which the distribution of HBV phases and HCV genotypes are provided worldwide 43, 44. Since recommendations for treatment initiation vary slightly between European, American, and Pacific liver associations, as well as WHO, applying epidemiological data to estimate the number of individuals needing treatment, and of them who is receiving treatment, is not straightforward. In fact, the WHO Global Hepatitis Report assesses the treatment gap for HBV as “unknown” while for HCV, it considers that all infected patients are in need of treatment 6. A more nuanced understanding of this step in the cascade‐of‐care is necessary.

#### 2.3.2 Novel treatments for chronic hepatitis B

With the success of DAA and along the lines of programmes aimed at HIV cure, there has been substantial momentum in developing therapeutic regimens to cure HBV 45. The major impediment to such a goal is the eradication of covalently‐closed circular DNA (ccc‐DNA), the template used to transcribe all viral proteins, which cannot be achieved with current NAs 46. Given that NAs only inhibit DNA polymerase activity, agents targeting other steps in the replication cycle are needed to further suppress intrahepatic replication. Development is underway of agents involved in blocking HBV entry via the sodium taurocholate co‐transporting polypeptide cell receptor (myristoylated pre‐S peptides, cyclophilin inhibitors), inhibiting gene expression at the mRNA level (short interfering RNAs), affecting core protein assembly (core protein allosteric modulators), and others (reviewed in 47). These agents are still in the early clinical trial phases and have yet to be assessed in HIV‐HBV co‐infected patients.

HBV infection is known to exert a wide range of immunological deficiencies, including, but certainly not limited to, depletion of virus‐specific cytotoxic T lymphocytes and increased T cell exhaustion 48. There is then active interest in developing agents that elicit or restore antiviral immunity. Current therapeutic strategies have been engineered to affect both innate (toll‐like receptor agonists) and adaptive (checkpoint inhibitors) arms of the immune system, but again are mostly experimental 47. Considering that these approaches may require a highly orchestrated immune response, as is the case with interferon‐based therapies 49, their efficacy could be reduced in immunocompromised HIV‐HBV co‐infected patients 50.

#### 2.3.3 Antiviral‐induced virological response and clinical outcomes

Concomitant reductions in HCC incidence are generally observed with effective HBV suppression from NA‐based therapy, yet this risk is not completely abrogated 51. A low proportion of treated individuals with adequate viral suppression do develop HCC during treatment, questioning the adequacy of current antiviral agents. HBV DNA viral loads or quantitative HBsAg are also poor predictors of these events and perhaps other replicative biomarkers could more accurately establish disease risk 52. Likewise, decreases in incident HCC have been observed in HIV‐HBV co‐infected patients over the past decade 53 as use of more potent anti‐HBV agents has increased, yet the implications of suppressed HBV DNA is unclear 54.

There are conflicting results on the risk of developing HCC after DAA‐induced SVR, with some studies reporting a higher HCC incidence rate 55, 56 and others, including a meta‐analysis, finding no or even a reduced rate 57, 58, 59. Compensated cirrhosis is a well‐known prognostic factor for HCC 60. Recent research on HCV‐induced epigenetic and transcriptional changes could further elucidate the mechanisms of developing HCC 61, but still require further study. Similarly, the consequences of HCV eradication on extrahepatic manifestations, such as diabetes or cardiovascular diseases, also need clarification. Data on DAA use and HCC incidence are starting to emerge in HIV‐HCV co‐infected patients and suggest that the risk of HCC may not be higher after HCV cure 62. Previous data from the pegylated‐interferon era have also suggested that co‐infected patients are not privy to HCC after SVR 63.

#### 2.3.4 Access to treatment for infected populations

Scaling‐up access to antiviral therapy has slowly begun in recent years with price negotiations, generic formulations, and localized production 6, 64. Still, potent anti‐HBV treatments, such as TDF, are limited to HIV‐HBV co‐infected patients in Sub‐Saharan Africa 24 or is not provided for more than a given amount of time. Cost of anti‐HCV treatments has been greatly reduced, allowing for more widespread access, but continue to pose financial burdens for some LMIC 65.

Severe clinical outcomes in individuals infected with viral hepatitis cannot, however, be reduced by treatment alone, but will require effective healthcare services. Many barriers in accessing these services currently exist 66, with many more studies on HCV than HBV‐infection 67. First, prioritization of distributing and reimbursing NAs and DAAs are based on constrained resources or little empirical evidence for some countries 6, 68, 69. Future research must aim to understand the epidemic of viral hepatitis, its disease progression and impact on quality of life in order to substantiate evidenced‐based policies. Second, some countries only allow specialists to prescribe DAAs, while many key populations have difficulty in accessing these services. Integrating HCV testing in healthcare services reaching key populations (HIV clinics, harm reduction, in‐prison services) and permitting community nurses or primary healthcare services to provide DAAs have shown increases in linkage to and retention in care 70, 71, 72. However, the impact of such strategies in other settings is potentially offset by other systemic and individual barriers. Negative perceptions of HCV treatment, penalization and stigmatization of drug use and, in some countries, sexual preferences are all social factors discouraging key populations from consulting healthcare services 7. Third, practitioners may be reluctant to treat individuals at high‐risk of reinfection. Rates of reinfection among persons who inject drugs are rather variable, but are mostly lower when enrolled in harm‐reduction programmes 73. Nevertheless, harm reduction services might not reach non‐opioid dependent persons and persons who formerly injected drugs, representing a major reservoir of infection 74. High reinfection incidence has been observed in HIV‐positive men who have sex with men, while the behavioural data to corroborate ongoing sexual risk behaviours as the underlying reason for this finding are scarce 75. Specific post‐treatment interventions aimed at reducing HCV re‐infected are strongly needed. Finally, few countries have established a national viral hepatitis plan 76, making it difficult to guide clinicians on the most appropriate line of care.

## 3 Conclusions

Notwithstanding the tools able to successfully prevent, suppress, and cure viral hepatitis, the most efficient way of their use requires considerable research. In the short‐term, HBV vaccine coverage should be increased to all regions of the world, while the reasons for reluctance to vaccinate could provide insights on accomplishing this goal. Cheaper and easier technology to test for viral hepatitis should be developed and used in campaigns to increase infection awareness in key populations. Treatment must be accessible to infected individuals to prevent disease progression. Finally, it is likely that a combination of these tools is required for elimination of viral hepatitis 7, 77. Although the WHO Global Hepatitis Report has clearly laid out the needs at each step of the viral hepatitis cascade‐of‐care, continuous and informed research could help prioritize these shifting needs for policy makers and stakeholders alike.

## Competing interests

AB has received research grants from the Agence nationale de recherche sur le sida et les hépatites virales (ANRS), SIDACTION, and personal fees from Gilead Sciences, Inc. LD has no conflict of interest to declare. KL has received research grants from the ANRS and SIDACTION; personal fees and non‐financial support from Gilead Sciences, Inc; personal fees and non‐financial support from Abbvie; personal fees from Janssen; grants, personal fees and non‐financial support from MSD.

## Authors’ contributions

AB and LD drafted the manuscript. KL gave critical revisions. All authors approved the final version of this manuscript.

## Funding

The authors report no funding in relation to the present commentary.
